# Efficient Visible-Light Photocatalytic Properties in Low-Temperature Bi-Nb-O System Photocatalysts

**DOI:** 10.1186/s11671-016-1583-6

**Published:** 2016-08-31

**Authors:** Haifa Zhai, Shuying Shang, Liuyang Zheng, Panpan Li, Haiqin Li, Hongying Luo, Jizhou Kong

**Affiliations:** 1 Henan Key Laboratory of Photovoltaic Materials, College of Physics and Materials Science, Henan Normal University, Xinxiang, 453007 People’s Republic of China; 2 National Laboratory of Solid State Microstructures, Nanjing University, Nanjing, 210093 People’s Republic of China; 3 College of Mechanical and Electrical Engineering, Nanjing University of Aeronautics and Astronautics, Nanjing, 210016 People’s Republic of China

**Keywords:** Bi-Nb-O photocatalyst, Citrate method, Visible-light photocatalytic property, Synergistic effect

## Abstract

Low-temperature Bi-Nb-O system photocatalysts were prepared by a citrate method using homemade water-soluble niobium precursors. The structures, morphologies, and optical properties of Bi-Nb-O system photocatalysts with different compositions were investigated deeply. All the Bi-Nb-O powders exhibit appreciably much higher photocatalytic efficiency of photo-degradation of methyl violet (MV), especially for Bi-Nb-O photocatalysts sintered at 750 °C (BNO750), only 1.5 h to completely decompose MV, and the obtained first-order rate constant (*k*) is 1.94/h. A larger degradation rate of Bi-Nb-O photocatalysts sintered at 550 °C (BNO550) can be attributed to the synergistic effect between *β*-BiNbO_4_ and Bi_5_Nb_3_O_15_. Bi_5_Nb_3_O_15_ with small particle size on *β*-BiNbO_4_ surface can effectively short the diffuse length of electron. BNO750 exhibits the best photocatalytic properties under visible-light irradiation, which can be attributed to its better crystallinity and the synergistic effect between *β*-BiNbO_4_ and *α*-BiNbO_4_. The small amount of *α*-BiNbO_4_ loading on surface of *β*-BiNbO_4_ can effectively improve the electron and hole segregation and migration. Holes are the main active species of Bi-Nb-O system photocatalysts in aqueous solution under visible-light irradiation.

## Background

Recent years, much attention has been focused on the environmental remediation due to the increasing pollution problems caused by the industries. Organic pollutants, particularly dyes, have a deleterious effect on human health [1]. In 1972, photosensitized decomposition of water into H_2_ and O_2_ using TiO_2_ semiconductor electrode was first reported by Fujishima and Honda [2]. Since then, a large number of semiconductor materials have been investigated as active catalysts for the reduction and/or elimination of environmental pollution in water and air due to their potential in the conversion of light energy. TiO_2_, as one of the most popular photocatalysts, can solely absorb the UV light, which accounts for only 4 % of the total sunlight. It greatly inhibits its practical applications for the decomposition of toxic and hazardous organic pollutants. Hence, it is very necessary to develop photocatalysts with high catalytic activities under the visible light. Currently, numerous strategies, such as doping [3–5], dye sensitization [6, 7], and growths of TiO_2_-based heterostructures [8, 9], have been developed, aiming to promote their photo-response performance to the visible range. Besides, other novel photocatalysts with outstanding visible-light photocatalytic properties have been developed, such as quantum dot-based photocatalysts [10–14].

Bismuth-based photocatalysts, due to their excellent photo-degradation performance for organic contaminant using visible light, have attracted much attention, such as BiWO_6_ [15], BiOX (X = Cl, Br, I) [16, 17], Bi_2_O_2_CO_3_ [18], BiNb_3_O_15_ [19], and BiNbO_4_ [20–25]. Among these materials, BiNbO_4_ is investigated for H_2_ generation and contaminant degradation under visible light, exhibiting greater photocatalytic performance than TiO_2_. In general, BiNbO_4_ has orthorhombic *α* and triclinic *β* phases; *α* phase synthesized at 900 °C irreversibly transforms to the high-temperature *β* phase (denoted as High-*β*) at 1020 °C [26]. Compared with *β* phase, the *α* phase always shows better photocatalytic performance due to the formation of a narrow conducting band and the electron and holes can effectively reach reaction sites on the surface in orthorhombic structure [27]. While in our former work, we first synthesized the pure low-temperature *β* phase (denoted as Low-*β*) at 700 °C and the visible-light photocatalytic performance test shows that the Low-*β* exhibits better photocatalytic efficiency compared with *α* phase [20, 28]. The formation of pure triclinic phase of BiNbO_4_ at low temperature can be attributed to the formation of intermediate Bi_5_Nb_3_O_15_ phase.

Compared with BiNbO_4_, the research of Bi_5_Nb_3_O_15_ as photocatalyst is rare, though it is expected to have high photocatalytic efficiency due to the composition of the conduction band (CB) and valance band (VB) same as BiNbO_4_ [29]. Because of the volatilization of Bi element at high temperature, the conventional solid state method requires critical control of Bi content to obtain stoichiometric Bi-Nb-O compounds; also, the resulting bulk products of several micrometers are harmful to efficient electronic diffusion, which inhibits the research and applications of Bi-Nb-O system photocatalysts. Citrate method, a simple way to obtain stable precursors and reactive, stoichiometric fine powders, has been widely used in the fabrication of various complicated oxides [30].

In this paper, low-temperature Bi-Nb-O system photocatalysts were prepared by the citrate method using homemade water-soluble niobium precursors. The structures, morphologies, optical properties of Bi-Nb-O system photocatalysts with different composition were investigated deeply. The visible-light photocatalytic properties were evaluated with the degradation of methyl violet (MV) under visible light irradiation. The synergistic effect between different compositions in Bi-Nb-O system was also proposed to explain the efficient visible-light photocatalytic properties.

## Methods

### Catalysts Preparation

Bismuth nitrate (Bi(NO_3_)_3_·5H_2_O), citric acid (CA), ammonia (NH_3_·H_2_O), and Nb-citrate (Nb-CA) aqueous solution were used as starting materials. The synthesis of water-soluble Nb-CA has been described in details in our previous work [31]. The Bi-Nb-O powders were prepared using the citrate method. Bi(NO_3_)_3_·5H_2_O was first dissolved in Nb-CA aqueous solution, followed by addition of CA. Then the solution was kept stirring at 60 °C, using ammonia to adjust the pH value to 7~8. Finally, the stable and transparent precursor solution was dried at 180 °C and then sintered at various temperatures from 500 to 800 °C for 3 h to obtain the Bi-Nb-O powders.

### Characterization

The structures of the Bi-Nb-O powders were characterized by X-ray diffraction (XRD; Rigaku-D/Max 2000) using Cu *K*α radiation. The scanning electron microscope (SEM; JSM-6700F) and transmission electron microscope (TEM; Tecnai F20 S-Twin, FEI) were used to examine the morphologies and grain sizes of the powders. The specific surface area was measured on a surface area apparatus (Micromeritics TriStar 3000, Shamidzu) at 77 K by N_2_ adsorption/desorption method (BET method). The photoluminescence (PL) spectra were detected using an F-280 fluorescence spectrophotometer with excitation wavelength of 320 nm. X-ray photoelectron spectroscopy (XPS) analysis was performed on Thermo Fisher K-Alpha equipment.

### Catalytic Tests

To evaluate the visible-light photocatalytic activities of Bi-Nb-O powders, the decomposition reaction of MV aqueous solution was carried out under irradiation of a 150-W Xe lamp (LA-410UV-3, Hayashi, Japan) at the natural pH value. The details have been described in the previous work and the photo-degradation process was monitored by an ultraviolet-visible near infrared (UV-vis-NIR) spectrophotometer (UV-3600, Shimadzu, Japan) [20]. The concentration of the residual MV in solution was determined as a function of irradiation time by measuring the maximum absorption at 582 nm.

To detect the active species during photocatalytic reactivity, hydroxyl radicals (·OH) and holes (h^+^) were investigated by adding 5 mM tert-butyl alcohol (*t*-BuOH; a quencher of ·OH) and EDTA-2Na (a quencher of h^+^) [32]. The method was similar to the former photocatalytic activity test with 30-min visible-light irradiation.

## Results and Discussion

**Fig. 1:**
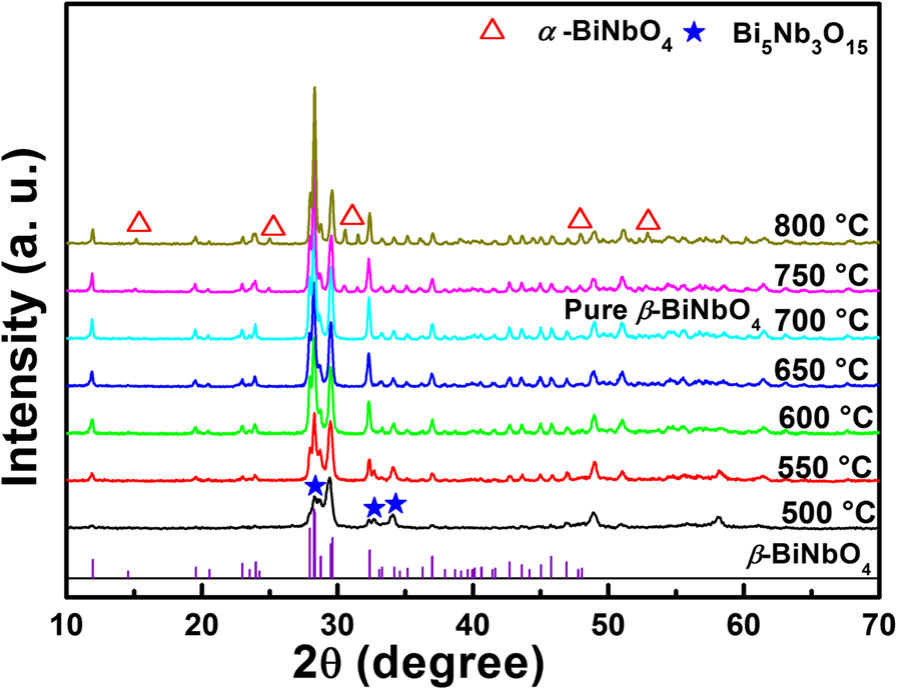
XRD patterns of Bi-Nb-O powders sintered at different temperatures for 3 h. ICSD pattern of *β*-BiNbO_4_ (JCPDF, No. 16-0486) is also inserted

Figure 1 shows the XRD patterns of Bi-Nb-O precursors sintered at different temperatures. At 500 °C, Bi_5_Nb_3_O_15_ appears as the major phase with other Bi-Nb-O compounds. With the sintering temperature increasing to 550 °C, most of the Bi_5_Nb_3_O_15_ decomposes and Low-*β* forms as the major phase. At 600 and 650 °C, only small amount of Bi_5_Nb_3_O_15_ remains. Pure *β* phase BiNbO_4_ is obtained at 700 °C; while with the further increase of temperature, *α* phase BiNbO_4_ forms and coexists with *β* phase even at 800 °C. The formation mechanism of Low-*β* and the phase transition from Low-*β* to *α* phase has been deeply discussed in our former work [28]. To concisely study the Bi-Nb-O system, the Bi-Nb-O photocatalysts prepared at 500, 550, 600, 650, 700, 750, and 800 °C were denoted as BNO500, BNO550, BNO600, BNO650, BNO700, BNO750, and BNO800 below, respectively.

**Fig. 2:**
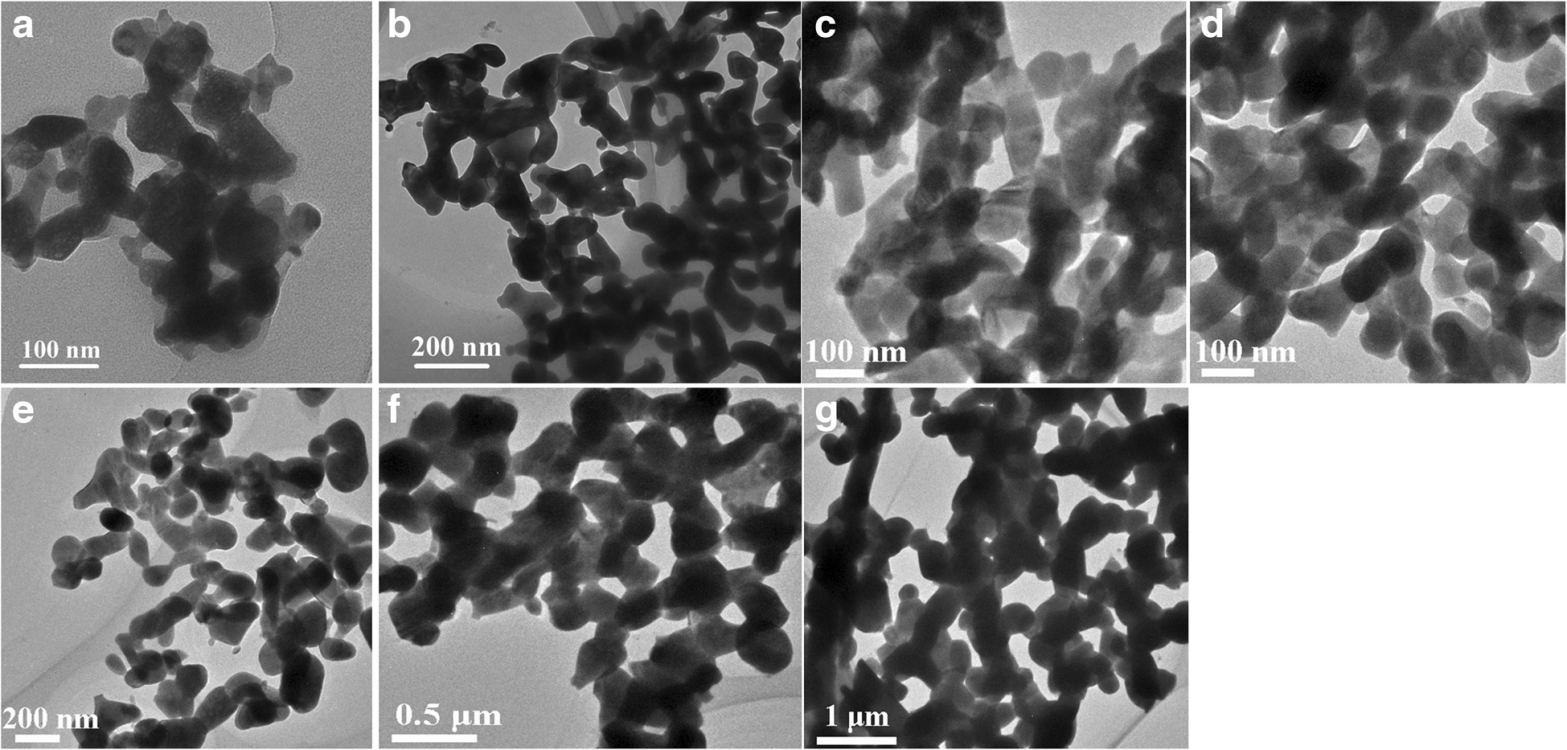
TEM images of Bi-Nb-O powders sintered at **a** 500 °C, **b** 550 °C, **c** 600 °C, **d** 650 °C, **e** 700 °C, **f** 750 °C, and **g** 800 °C for 3 h

The TEM images of Bi-Nb-O powders sintered at different temperatures are given in Fig. 2. As seen in the figure, the higher the sintering temperature, the larger the grain size becomes. For BNO500, the grain size is about 30–40 nm, while for BNO800, it is about 300 nm; also, the shape of Bi-Nb-O powders seems irregular. Table 1 summarizes the grain size and specific areas of Bi-Nb-O powders sintered at various temperatures. The specific areas of the Bi-Nb-O powders are comparable with each other, except for BNO800 with the largest grain size. For BNO700, the specific surface area is 12.2 m^2^/g. It seems that the Bi-Nb-O powders prepared by the citrate method show larger specific surface area than other groups’ results [33].

**Table 1:** The grain sizes, specific surface areas, and UV-vis absorption data of Bi-Nb-O powders sintered at different temperatures

Catalysts	BNO500	BNO550	BNO600	BNO650	BNO700	BNO750	BNO800
Grain size (nm)	30~40	~60	70~80	~100	100~200	~200	300~400
Specific surface areas (m^2^/g)	9.7	11.2	10.5	10.3	12.2	11.6	7.9
Band gap (eV)	2.88	2.83	2.89	2.88	2.78	2.88	2.91
*λ* ^a^ (nm)	431	439	429	431	447	431	427

^a^Maximum absorptive wavelength is estimated from the intercept of the tangents to the plots

**Fig. 3:**
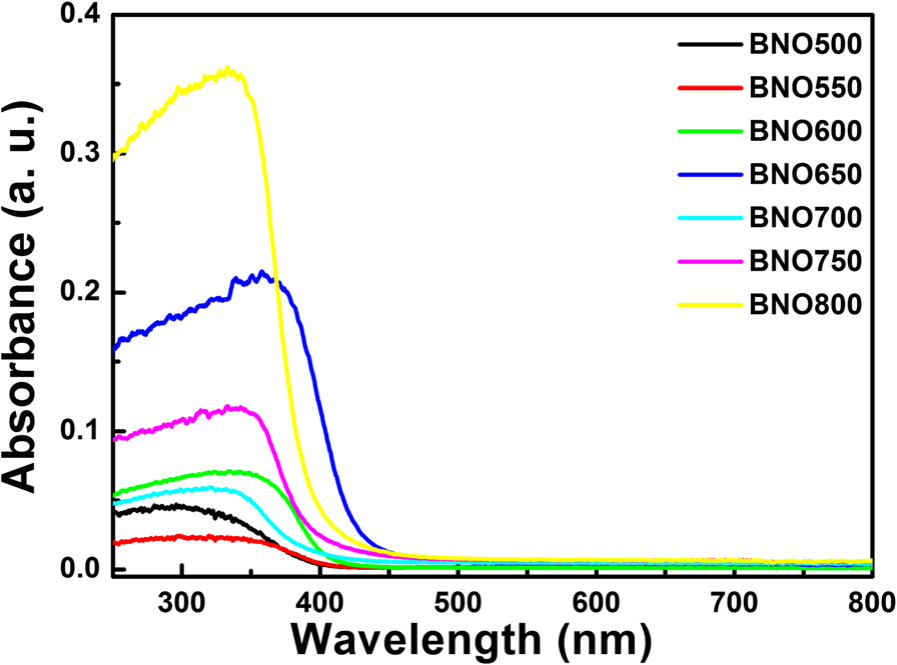
UV-vis diffuse reflectance absorbance spectra of Bi-Nb-O powders

Figure 3 shows the UV-vis diffuse reflectance absorbance spectra of Bi-Nb-O powders. The absorbance coefficient (*α*) is transformed from the diffuse reflection spectra based on the Kubelka-Munk (K-M) theory using pressed BaSO_4_ powders as a reference. The relation between the absorption edge and the incident photon (*hv*) can be written as follows:

1 $$ \alpha hv=A{\left(hv-{E}_g\right)}^n $$

where *A* is the band edge constant and *n* is an index which assumes the values 1/2 and 2 for direct allowed and indirect allowed transitions, respectively. Because Low-*β* as the major phase in Bi-Nb-O system is the indirect band gap semiconductor, the value of *n* is taken as 2. The energy band gaps of Bi-Nb-O powders are estimated, as listed in Table 1. The energy band gap is consistent with that obtained using density functional theory computation [34]. The band gaps of Bi-Nb-O powders suggest all of them have the visible-light photocatalytic performance through direct photo-absorption, and the critical absorbance wavelength is above 400 nm. The color of the powders is pale yellow, which is consistent with the band gaps.

**Fig. 4:**
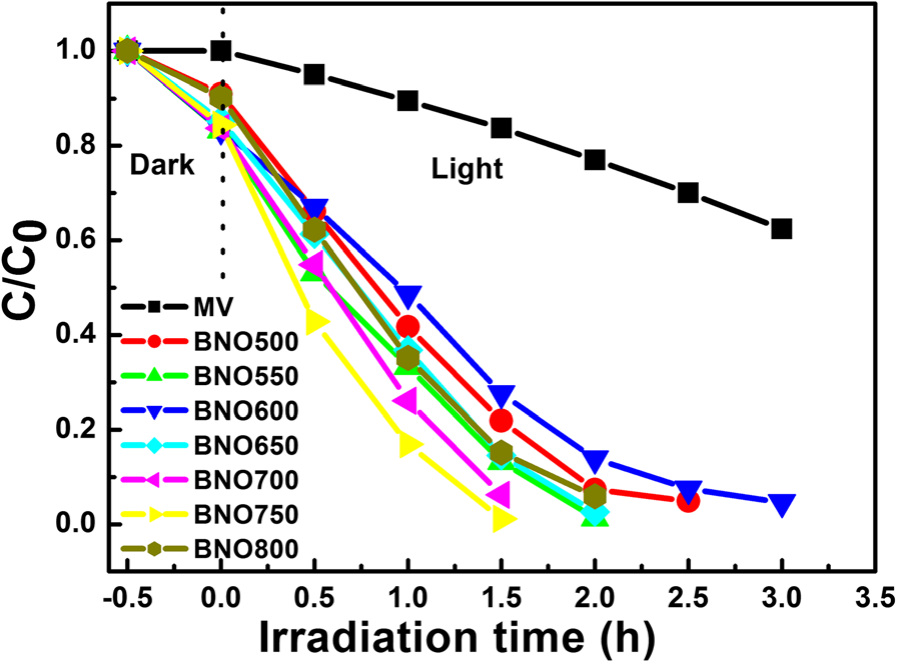
Photo-degradation of MV with respect to the irradiation time using Bi-Nb-O powders exposed to visible light. Adsorption ability of Bi-Nb-O powders is tested after stirring for 1 h in the absence of light to achieve the equilibrium adsorption

The photocatalytic activities of Bi-Nb-O powders are evaluated via photo-degradation of MV under visible-light irradiation, as shown in Fig. 4. In the experiment, the degradation of MV without photocatalyst is also studied as a reference. The dashed line in Fig. 4 represents the MV concentration after adsorption/desorption equilibrium. All the Bi-Nb-O powders show good adsorption ability, about 16 % for most catalysts except for BNO500 and BNO800 with 9 %. The adsorption ability of MV is an important factor to decompose MV in the photo-degradation process. Compared with the degradation of MV without catalyst, all the Bi-Nb-O powders exhibit appreciably much higher photocatalytic efficiency of photo-degradation of MV, especially for BNO750, only 1.5 h to completely decompose MV. It shows that low-temperature Bi-Nb-O photocatalysts have efficient visible-light photo-degradation properties. For Bi-Nb-O photocatalysts, the degradation mechanism of MV under visible-light irradiation involves photocatalytic and photosensitization pathways, and the latter has a dominant role in the degradation [20]. They can not only absorb the visible light with the wavelength short enough to activate the electron and hole segregation directly but also use the visible light indirectly with the absorbed MV molecules acting as antennae on photocatalysts to absorb the light. The photocatalytic efficiency of Bi-Nb-O catalysts is ranked in an order from the highest to the lowest: BNO750 > BNO700 > BNO550 > BNO650 > BNO800 > BNO500 > BNO600. Interestingly, there exist two peaks in Bi-Nb-O system. BNO550 shows a larger degradation rate than BNO500, BNO600, and BNO650; at the same time, BNO750 has the best degradation properties than others.

**Fig. 5:**
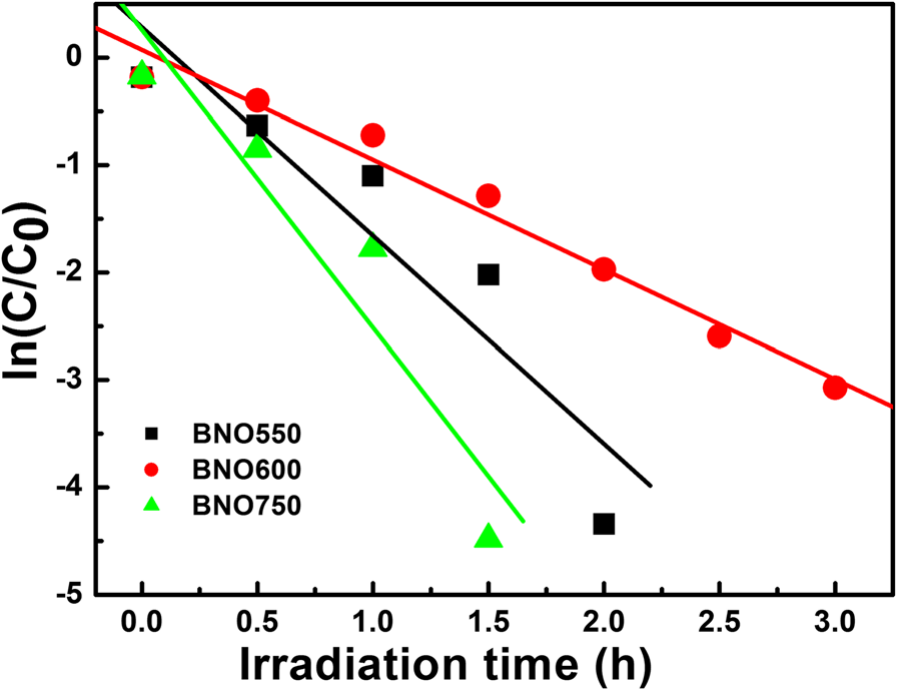
Kinetic fit for the photo-degradation of MV in the presence of BNO550, BNO600 and BNO750 powders, respectively

For the photo-degradation of MV, it is found that the degradation using Bi-Nb-O catalysts obeys the pseudo-first-order kinetics, described by the modified Langmuir-Hinshelwood kinetics model [35]. The representation is given as follows:

2 $$ \ln \left({C}_0/C\right) = kt $$

where *C* is the concentration of MV solution, *t* is the reaction time, and *k* is the constant of the pseudo-first-order rate. Plots of ln(*C*_0_/*C*) versus irradiation time for the degradation of MV using BNO550, BNO600, and BNO750 as catalysts is shown in Fig. 5. The obtained first-order rate constants (*k*) are 1.94, 1.02, and 2.77/h; the apparent rate constant of BNO750 is about 2.7 times higher than that of BNO600. So the degradation rate of MV with BNO750 is much higher than that with BNO600.

As for Bi-Nb-O compounds, no matter Bi_5_Nb_3_O_15_ or *α*, *β* phase BiNbO_4_, their electronic structures are composed of VB by O 2p state and CB by hybridization states of Bi 4p, Nb 4d, and O 2p [36]. In other group’s research, Bi_5_Nb_3_O_15_ powder with a small size has higher photocatalytic activity than P25 and bulk *α* phase BiNbO_4_, due to short diffuse length of electron and efficient visible-light harvesting [19]. In our Bi-Nb-O system, Bi_5_Nb_3_O_15_ phase was first formed with a smaller particle size at 500 °C. With the sintering temperature increase, the major phase Bi_5_Nb_3_O_15_ decompose, which results in Low-*β* phase BiNbO_4_ coexisting with a smaller amount of Bi_5_Nb_3_O_15_ particles in BNO550. The better photocatalytic property of BNO550 can be attributed to the synergistic effect between *β*-BiNbO_4_ and Bi_5_Nb_3_O_15_. Bi_5_Nb_3_O_15_ with a smaller particle size on *β*-BiNbO_4_ surface can effectively short the diffuse length of electron, subsequently decreasing the recombination rate of electrons and holes. When the sintering temperature is above 600 °C, the content of Bi_5_Nb_3_O_15_ is negligible and the photocatalytic properties are mainly from Low-*β* phase BiNbO_4_.

Compared with the High-*β*, the crystal evolution of Low-*β* has not completed yet. The photocatalytic properties are promoted with the sintering temperature, which means better grain crystallinity. As in Fig. 4, the adsorption test in “dark” shows that the adsorption ability is nearly the same for BNO powders sintered between 600 and 750 °C. So the improved grain crystallinity has the dominant role in the promotion of photocatalytic properties. As the sintering temperature increase to 750 °C, the major phase is Low-*β* phase BiNbO_4_ coexists with a smaller amount of *α* phase BiNbO_4_. For BNO800, the content of *α* phase increases and the adsorption ability decreases, as shown in Fig. 4. As described in our former work, Low-*β* phase BiNbO_4_ prepared by the citrate method has better photocatalytic performance than *α* phase BiNbO_4_ [20]. BNO750 having the best photocatalytic properties under visible light may be attributed to two aspects: one is the better crystallinity and the other is the synergistic effect between Low-*β* phase BiNbO_4_ and *α* phase BiNbO_4_. Compared with *β* phase, the *α* phase with [NbO_4_] chains favors the formation of a narrow conducting band and the electrons and holes can effectively reach reaction sites of photocatalysts [27]. For BNO750, the small amount of *α* phase BiNbO_4_ loading on the surface of Low-*β* phase BiNbO_4_ can effectively improve the electron and hole segregation and migration. So BNO750 exhibits the best visible-light photocatalytic properties in low-temperature Bi-Nb-O system photocatalysts.

**Fig. 6:**
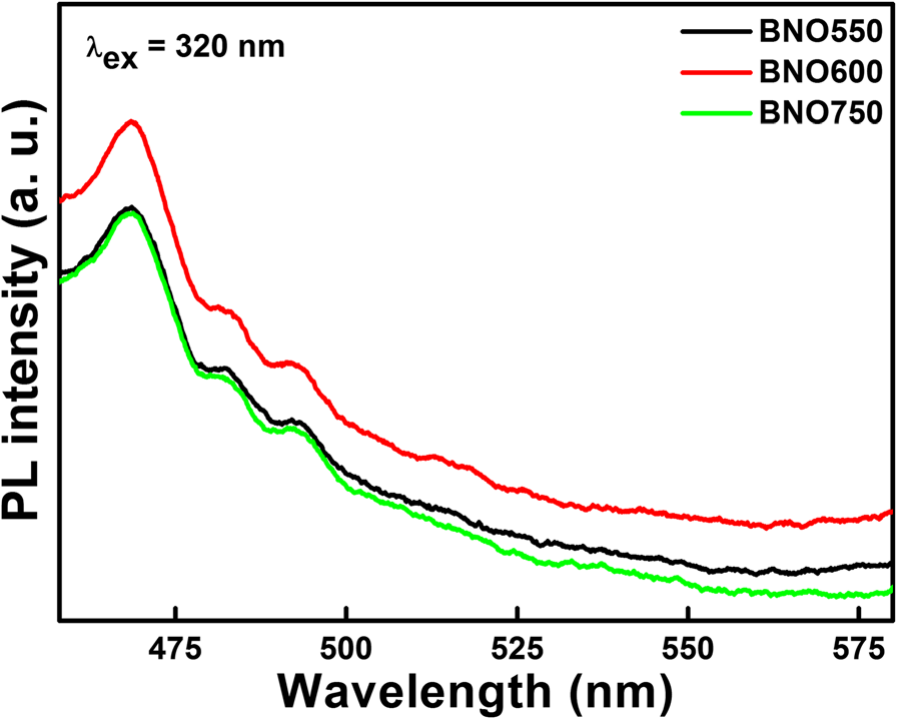
Room temperature PL spectra of BNO550, BNO600, and BNO750 catalysts

The better separation of photo-generated electrons and holes in BNO550 and BNO750 catalysts is confirmed by PL spectra, as shown in Fig. 6. As we know, PL emission spectra mainly result from the recombination of free carriers; therefore, PL spectra measurement is an effective method to survey the separation efficiency of the photo-generated charge carriers in semiconductors [37]. It can be seen that compared with BNO600, BNO550 and BNO750 have smaller emitting peaks around 468 nm, which means they have longer charge carriers lifetime and improved efficiency of interfacial charge transfer, and then enhanced photocatalytic activity, well consistent with Fig. 4.

**Fig. 7:**
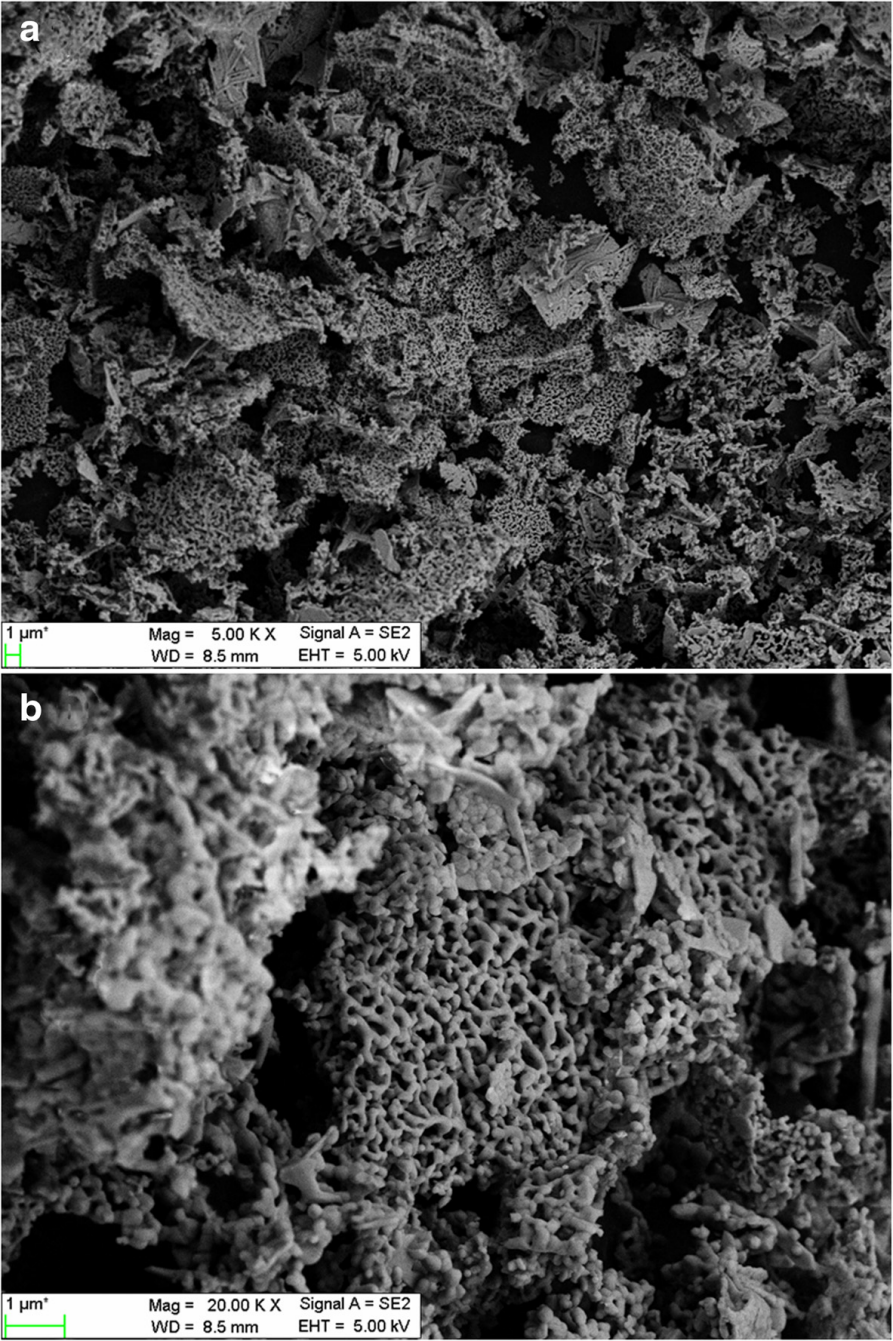
SEM images of BNO750 with magnifications of **a** ×5000 and **b** ×20,000

As discussed above, BNO750 exhibits the best photocatalytic performance among low-temperature Bi-Nb-O system photocatalysts. The morphology of BNO750 powders is investigated using SEM, as shown in Fig. 7. It can be seen that BNO750 powders is porous and the particles connect with each other to form strips, which can be regarded as a honeycomb structure. The honeycomb structure is beneficial to photo-degradation of MV due to large specific areas.

**Fig. 8:**
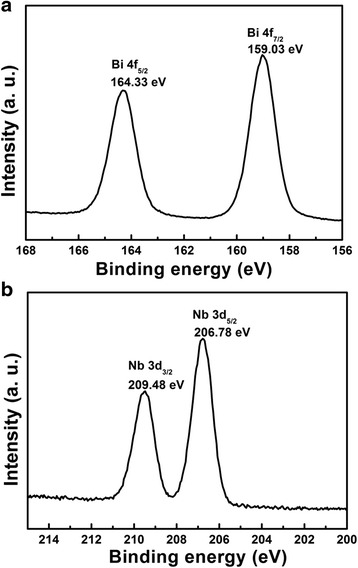
XPS spectra of **a** Bi 4f and **b** Nb 3d for BNO750 photocatalysts

The chemical component of BNO750 catalyst is characterized using XPS, as shown in Fig. 8. The peaks at 164.33 and 159.03 eV correspond to Bi4f_5/2_ and Bi4f_7/2_, respectively, and these peaks confirm the presence of Bi^3+^ in BNO750 lattice. At the same time, the peaks of Nb3d_3/2_ and Nb3d_5/2_ in Fig. 8b confirm the presence of Nb^5+^. There is no other valence state observed in Fig. 8, which means no metallic bismuth or reduced Nb oxide species formed in BNO750 [23].

**Fig. 9:**
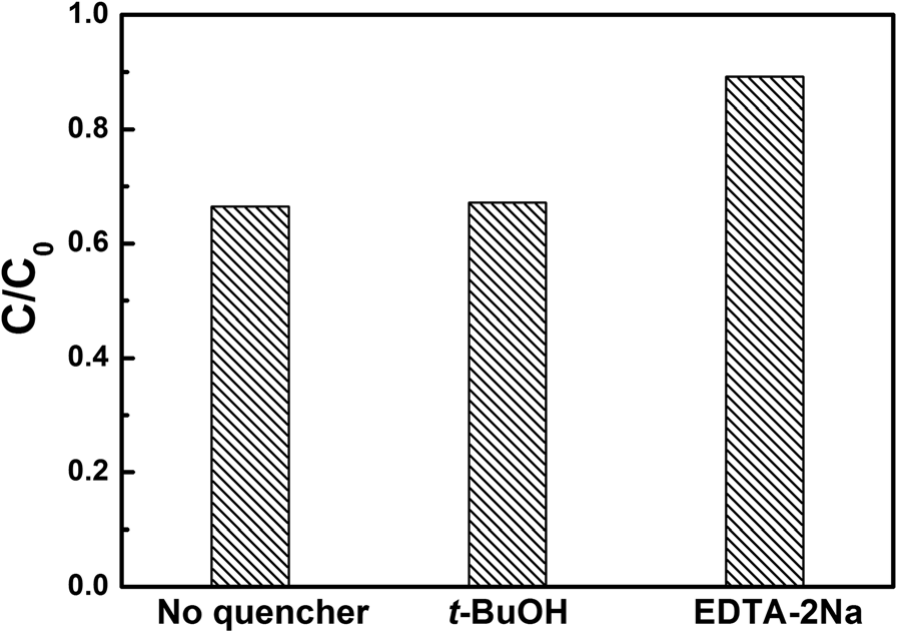
Trapping experiment of active species during the degradation of MV under visible-light irradiation with the presence of BNO750 catalysts

Figure 9 displays the trapping experiment of active species during the photocatalytic reaction process with BNO750 catalysts. It can be seen that the degradation of MV is not affected by the addition of *t*-BuOH, while the degradation rate lowers obviously with the addition of EDTA-2Na. Therefore, it can be concluded that holes are the main active species of Bi-Nb-O system photocatalysts in aqueous solution under visible-light irradiation, rather than ·OH.

The effect of operating parameters such as the amount of catalyst loading, pH value, and the additive H_2_O_2_ concentration on the photocatalytic performance of low-temperature Bi-Nb-O photocatalysts has been investigated also, similar to that of pure Low-*β* phase BiNbO_4_ described in our former work [20]. It shows that the optimal operation conditions are catalyst loading of 1 g/L, pH value of 8, and the additive H_2_O_2_ concentration of 2 mmol/L.

## Conclusions

Bi-Nb-O system photocatalysts were prepared by the citrate method using homemade water-soluble niobium precursors. The structures, morphologies, and optical properties of Bi-Nb-O system photocatalysts with different compositions were investigated deeply. All the Bi-Nb-O powders exhibit appreciably much higher photocatalytic efficiency of photo-degradation of MV, especially for BNO750, only 1.5 h to completely decompose MV, and the obtained *k* is 1.94/h. Larger degradation rate of BNO550 can be attributed to the synergistic effect between *β*-BiNbO_4_ and Bi_5_Nb_3_O_15_. Bi_5_Nb_3_O_15_ with a smaller particle size on *β*-BiNbO_4_ surface can effectively short the diffuse length of electrons. BNO750 exhibits the best photocatalytic properties under visible-light irradiation, which can be attributed to its better crystallinity and the synergistic effect between *β*-BiNbO_4_ and *α*-BiNbO_4_. The small amount of *α* phase BiNbO_4_ loading on surface of Low-*β* phase BiNbO_4_ can effectively improve the electrons and holes segregation and migration. Holes are the main active species of Bi-Nb-O system photocatalysts in aqueous solution under visible-light irradiation.
